# Expected value of the additional state in evaluating the method of quantification and uncertainty of additional states in an analytical model of grade I hypertension

**DOI:** 10.1186/s40780-014-0006-z

**Published:** 2015-01-28

**Authors:** Takeshi Uchikura, Makoto Kobayashi, Masayuki Hashiguchi, Mayumi Mochizuki

**Affiliations:** Division for Evaluation and Analysis of Drug Information, Faculty of Pharmacy, Keio University, 1-5-30 Shibakoen, Minato-ku, Tokyo, 105-8512 Japan; Department of Hospital Pharmaceutics, Showa University School of Pharmacy, 1-5-8 Hatanodai, Shinagawa-ku, Tokyo, 142-8555 Japan; CRECON Medical Assessment Inc, 2-12-15 Shibuya, Shibuya-ku, Tokyo, 150-0002 Japan

**Keywords:** Expected Value of the Additional State, Pharmacoeconomics, Hypertension, Quantification, Uncertainty

## Abstract

**Background:**

In the construction of pharmacoeoconomic models, simplicity is desirable for transparency (people can see how the model is built), ease of analysis, validation (how well the model reproduces reality), and description. Few reports have described concrete methods for constructing simpler models. Therefore we focused on the value of additional states and uncertainty in disease models with multiple complications.

**Objectives:**

The objective of this study was to examine the possibility of ranking additional states in disease models with multiple complications using a method for evaluating the quantification and uncertainty of additional states.

**Methods:**

The expected value of additional states (EVAS) was formulated to calculate the value of additional states from the variation between analytic models using the net benefit method, and uncertainty was subtracted from the variation. We also verified the usefulness and availability of this method in grade I hypertension as a verification of the disease model. We assumed that stroke was recognized as an associated complication of hypertension in the basic model. In addition, stroke recurrence, coronary heart disease (CHD), and end-stage renal disease (ESRD) were assumed to represent other complications of hypertension. Ten thousand Monte Carlo simulations were performed, and the probability distribution was assumed to be the beta distribution in clinical parameters. The ranges of clinical parameters were ±6.25%, 12.5%, 25%, and 50% of the standard deviation from the mean value.

**Results:**

The EVAS in complications of CHD showed the greatest uncertainty. In contrast, the EVAS of ESRD differed from stroke recurrence in the value ranking by uncertainty.

**Conclusions:**

The EVAS has the potential to determine the ranking of additional states based on the quantitative value and uncertainty in disease models with multiple complications.

**Electronic supplementary material:**

The online version of this article (doi:10.1186/s40780-014-0006-z) contains supplementary material, which is available to authorized users.

## Background

Pharmacoeoconomic model analysis is conducted to combine various parameters (effect, side effects, quality of life [QOL], and cost data) using modeling techniques, e.g., the Markov model [1]. The construction of analytic models is a key issue affecting their results. The guidelines for modeling analysis refer to internal validity, e.g., the course of a disease or clinical process, and external validity, e.g., comparing model results with real-world results [2,3]. Thus, The Joint Report of the International Society for Pharmacoeconomics and Outcomes Research and the Society for Medical Decision Making (report of the ISPOR-SMDM Modeling Good Research Practices Task Force-2 [4]) mentions that the simplicity of models is desirable for ease of analysis, description, transparency (people can see how the model is built), and validation (how well the model reproduces reality) [5]. However, few reports have described concrete methods for constructing simpler models.

Generally, in the construction of analytical (disease) models, we assume that multiple complications may occur as the outcome of a specific disease. The complications in the analytical model are selected based on their importance (generally referred to as “a state”), but currently there is no evaluation method for that purpose. Furthermore, it is important to consider the influence of the uncertainty of the state itself, but that is not done at present. Therefore, if we could attempt to estimate the quantitative value of additional states in an analytical model and examine the possibility of ranking additional states in disease models with multiple complications, it would contribute to the establishment of useful analytical models. Therefore, in this study we attempted to estimate the quantitative value of additional states and rank those additional states, in addition to evaluating the robustness of ranking while considering uncertainty.

## Objectives

The objective of this study was to examine the possibility of ranking additional states in disease models with multiple complications using a method for evaluating the quantification and uncertainty of additional states.

## Methods

### Method to evaluate quantification of the value of additional states and uncertainty of transition probabilities

We applied the net benefit method [6] to estimate the quantification of the value of additional states. This method assumes a net monetary benefit and a net health benefit in the same index using the threshold (e.g., 1 quality-adjusted life year [QALY] = 5 million yen) of an incremental cost-effectiveness ratio, and evaluates intervention effects in the single index as the result of cost-effectiveness analysis. This evaluation converts costs and QALY into the same index using a threshold (λ) by applying the concept of net benefit and subsequently adds these values as components of the value of the states. Specifically, analytical model A is is the basic model, and analytical model B reflects the addition of a specific complication as an additional state to analytical model A. We estimated the value of the additional state as the change in the cost and effectiveness *for the net benefit* between the two models using Eq. (1):

1 $$ \mathrm{Value}\ \mathrm{of}\ \mathrm{additional}\ \mathrm{state} = \left({\mathrm{C}}_{\mathrm{B}}\hbox{--} {\mathrm{C}}_{\mathrm{A}}\right)/\uplambda + \left({\mathrm{E}}_{\mathrm{A}}\hbox{--} {\mathrm{E}}_{\mathrm{B}}\right)\cdot \cdot \cdot \cdot $$

where C_A_ = the cost of analytical model A, C_B_ = the cost of analytical model B, λ = 5,000,000 yen (the value of 1 QALY), E_A_ = the effectiveness (QALY) of analytical model A, and E_B_ = the effectiveness (QALY) of analytical model B.

Then, we defined the expected value of the additional state (EVAS) as the true value of the additional state, taking into account the robustness of the results with the uncertainty of the occurrence of the additional state, and estimated the value using Eq. (2). The uncertainty of the additional state based on the transition probabilities is calculated as the absolute value of the difference in the value of the additional state and a trial value in Monte Carlo simulations. Monte Carlo simulations are recognized as a useful method for performing multiple simulations of a model to obtain stable estimates of its variability [1,7]. In addition, the cumulative values were obtained from 10,000 Monte Carlo simulations of “the value of the additional state” and “the uncertainty of additional state.”

2 $$ \mathrm{EVAS} = \mathrm{value}\ \mathrm{of}\ \mathrm{the}\ \mathrm{additional}\ \mathrm{state}\ \hbox{--}\ \mathrm{uncertainty}\ \mathrm{of}\ \mathrm{that}\ \mathrm{additional}\ \mathrm{state}\cdot \cdot \cdot \cdot $$

Using Eq. (1), we can calculate each value of multiple complications (states) assumed in a specific disease based on costs and QALY and rank these complications (states) in the analytical model. Furthermore, we can estimate the robustness of the results based on the uncertainty of each state itself by estimating the EVAS from Eq. (2). Therefore, we can select a specific complication among multiple complications of a disease, because this method ranks multiple complications that consider the values of additional states and the robustness of the value of uncertainty of additional states.

### Disease model used for examining the ranking of the values of additional states

**Figure 1:**
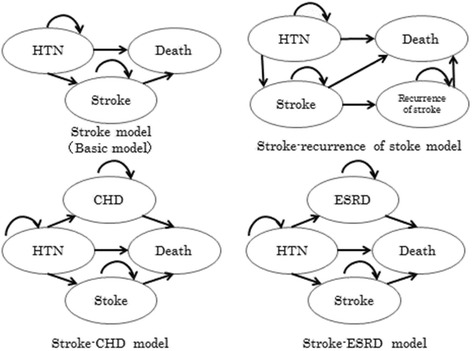
**The basic (stroke) model and each analytical model that assumed an additional state (complication).** HTN: hypertension, CHD: coronary heart disease, ESRD: end-stage renal disease.

We examined this evaluation method in hypertension, because it is a chronic model known to be associated with multiple complications. The basic model referred to the Markov model of grade I hypertension reported previously [8]. We did not assume an intervention effect for hypertension, because this model is less for pharmacoeconomic evaluation than for determining the value of additional states. The basic model covers stroke that is recognized as associated with hypertension [9]. In addition, stroke recurrence, coronary heart disease (CHD), and end-stage renal disease (ESRD) were assumed to represent other complications resulting from hypertension (Figure 1).

### Transition probabilities, QOL, and cost data on states in the disease model

The hypothetical patients were assumed to be 55-year-old men, and the analytical time line was 10 years, because the Japan Public Health Center (JPHC) study [10] of external validity enrolled men with grade I hypertension who had an average age of 56.0 years and the follow-up period was 11.0 years. We also used the annual incidences of stroke and CHD in patients with grade I hypertension (1000 persons/year) reported in the JPHC study [10]. The incidence of ESRD was also used as epidemiologic data in patients with grade I hypertension [11]. The rate of stroke recurrence and mortality after stroke, CHD, and ESRD were calculated from Japanese epidemiologic data [12-16]. The fifth-year parameter of stroke and the first-year parameter of mortality from CHD were used for the subsequent year and thereafter (Table 1). Nonstroke mortality was calculated based on the death rate of the Japanese population stratified by age [17].

**Table 1:** **Clinical parameters**

	**Mean**
Annual incidence of stroke in grade I hypertension (1000 people/year)	
Men [10]	4.9
Annual incidence of CHD in grade I hypertension (1000 people/year)	
Men [10]	1.17
Annual incidence of ESRD in grade I hypertension (1000 people/year)	
Men [11]	0.21
Recurrence rate of stroke [12]*	
First year	12.9%
Second to fifth year	8.2%
Mortality after stroke [13]*†	
First year	20.7%
Second year	6.7%
Third year	5.8%
Fourth year	5.9%
Fifth year	5.9%
Mortality after CHD [14,15]	
In hospital	7%
One year (1000 people/year)	20.4
Mortality after ESRD [16]	
One year	9.7%

*Sixth year and thereafter use the same parameter as the fifth year.

^†^Mortality after recurrence of stroke uses the same parameter.

The degree of disability after stroke was scored using the modified Rankin Scale (mRS) [18,19], and allocations of patients were derived from the proportion of stroke type and mRS score at hospital discharge [20,21]. The degree of disability was assumed to remain constant over time.

Utilities for patients with disability after stroke and ESRD were based on Japanese data [22,23] and those after CHD were based on non-Japanese (UK) data [24] The utilities of health (hypertension alone) and death were assumed to be 1 and 0, respectively (Table 2).

**Table 2:** **Utility in patients after stroke, CHD, and ESRD** [22-24]

	**mRS 0** *	**mRS 1**	**mRS 2**	**mRS 3**	**mRS 4**	**mRS 5**	**Death**
Stroke	1*	0.83	0.67	0.45	0.24	0.09	0*
CHD†	First year: 0.68, second year or later: 0.72						
ESRD	0.75						

*Assumed in this analysis.

†Data obtained from acute MI patients.

The cost of stroke treatment in the acute phase was obtained from the average number of hospitalized days based on the highest number of diagnostic procedure combinations in patients with each type of stroke [25]. The cost of recovery-phase rehabilitation facility care was calculated based on an average of 88 hospitalized days [26]. The annual cost of care was estimated from the amount of maximum payment for the categories of allowances for nursing care per month [27], which correspond to mRS scores of 2–5, and it was calculated until death. In addition, we calculated work lost until the age of 65 years if the patients died at age 64 years or younger.

The cost of CHD treatment for myocardial infarction (MI) in the acute phase was obtained from actual hospitalization costs (an average of 25 hospitalized days) [28]. The costs of nursing care and work lost (excluding hospitalization in the acute phase) were not included, because CHD has relatively little affect on physical functions. The cost of ESRD treatment was assumed to be represented by that for dialysis [23] and conservative management [29] (Table 3; see Additional file 1).

**Table 3:** **Categorization of assumed costs related to medical treatment and others after stroke, CHD, and ESRD**

		**Severity**	**Acute-phase hospital care cost**	**Recovery-phase care cost**	**Ambulant treatment cost**	**Nursing care cost**	Work lost*
After stroke	First year	mRS0-1	●		●		●
		mRS2-5	●	●	●	●	●
	Second year or later	mRS0-1			●		● (for clinic visits)
		mRS2-5			●	●	●
After CHD	First year	-	●		●		● (in hospital)
	Second year or later	-			●		● (for clinic visits)
After ESRD	-	-			● (hemodialysis or conservative management)		● (for clinic visit)

The data on medical costs were obtained from official prices in Japan [25,30,31]. The costs of work loss reflected the average wages of each age cohort for regular employees [32].

### Discounting

This study assumed that the discount rate was 3% and 0% (no discounts) for reference values.

### External validity

The external validity of analytical models compared the overall mortality from grade I hypertension patents in the JPHC Study [10] with the overall mortality of each analytical model.

### Statistical analyses

Ten thousand Monte Carlo simulations were performed for each evaluation. The probability distribution for transition probabilities was assumed to be the beta distribution [33]. The ranges of transition probabilities were ±6.25%, 12.5%, 25%, and 50% of the standard deviation (SD) from the mean. The simulation software TreeAge2013 (TreeAge Software, Inc. USA) was used.

## Results

### Evaluation of external validity

The overall mortality rate per year from the JPHC Study [10] was 0.0084, in the basic model (stroke alone) it was 0.0086, in the stroke-recurrence model it was 0.0087, in the stroke-CHD model it was 0.0087, and in the stroke-ESRD model it was 0.0086.

### Evaluation of the value of additional states

The values of additional states (complications) in the grade I hypertension model were 116 (92 for cost) for stroke recurrence, 295 (122 for cost) for CHD, 137 (100 for cost) for ESRD with dialysis, and 53 (16 for cost) for ESRD with conservative management (units are QALY). No differing trend was seen when no discount was assumed (Table 4).

**Table 4:** **Expected value of additional state (EVAS) of each complication**

	Value of additional state*	Uncertainty**	EVAS^†^
		(±6.25% SD)	
		(±12.5% SD)	
		(±25% SD)	
		(±50% SD)	
Recurrence of stroke	116 (140)	7 (8)	109 (132)
		13 (16)	102 (124)
		26 (32)	89 (108)
		52 (60)	64 (80)
CHD	295 (356)	15 (18)	280 (338)
		30 (36)	265 (320)
		60 (72)	235 (284)
		117 (142)	178 (214)
ESRD: Dialysis treatment	137 (167)	7 (8)	130 (159)
		14 (16)	123 (151)
		27 (33)	120 (134)
		53 (64)	84 (103)
Conservative treatment	53 (65)	3 (4)	50 (61)
		6 (7)	47 (58)
		12 (15)	41 (50)
		23 (28)	30 (37)

Cumulative values of 10,000 Monte Carlo simulations.

CHD: coronary heart disease, ESRD: end-stage renal disease.

*The value of additional state = (C_B_–C_A_)/λ+ (E_A_–E_B_).

**Uncertainty = an absolute value of the difference of the value of the additional state (average) and the trial value of the Monte Carlo simulations.

^†^EVAS = the value of the additional state – the uncertainty (of the additional state).

Figures in parentheses indicate values when the discount rate is 0%.

### Evaluation of the EVAS

The EVAS when uncertainty was considered tended to become smaller if the variable of the transition probabilities of the state grew larger. However, the ranking of additional states did not differ markedly when uncertainty was not taken into consideration. Therefore, the EVAS of CHD was the greatest of the additional states examined, even when the SD was ±50%, and the EVAS of ESRD with conservative management was the least in each case. In contrast, the EVAS of ESRD with dialysis was greater than that for stroke recurrence when uncertainty was not considered and reached 84 when the SD was ±50%. This estimate was less than the 89 for the EVAS of stroke recurrence when the SD was ±25% (Table 4).

## Discussion

In an attempt to construct a simpler model, this study ranked the importance of multiple complications in hypertension to estimate the value of additional states in a quantitative analytical model including transition probabilities, QOL, and cost data. In addition, we evaluated the robustness of the ranking of these values when considering the uncertainty of transition probabilities for the EVAS. To the best of our knowledge, this was the first experiment on quantification and ranking of the value of states along with uncertainty.

The ranking of the value of additional states was in the order CHD, ESRD with dialysis, stroke recurrence, and ESRD with conservative management in the analytical model of 55-year-old men with grade I hypertension. The differences in the assumed treatment in ESRD influenced the ranking of the value of the additional state, because the cost of dialysis treatment is 100, but that of conservative treatment is 16 (the value of QALY conversion). Thus, the ranking of ESRD with dialysis and stroke recurrence will change with an assumption of the uncertainty of transition probabilities of the EVAS.

In the development of analytical models, the difference in internal validity and results of analytical models must be weighed [34,35], particularly in analytical models constructed by academic groups in competitions like the Mount Hood Challenge Meeting [36]. Few reports referred to concrete methods for constructing simpler models. If our method enables the ranking of multiple complications in a specific model based on analytical values, more important and robust additional states could be identified. This would contribute to the development of a simpler model for pharmacoeconomic evaluation.

The limitations of this study were that: 1) only grade I hypertension was examined; 2) the setting of the threshold (λ) in this method could be refined; and 3) the variation in all transition parameters was assumed to have the same range in a state. Although only grade I hypertension was considered, Eq. (2) shows that there would be no difference in the ranking of the value of the state based on the EVAS in other diseases. Therefore, this method has sufficient potential to be widely applied. The threshold (λ) was set at 5 million yen for 1 QALY because 1 QALY in Japan was reported to be between approximately 5 and 6 million yen [37]. However, care must be taken because the results (the value of states) will change based on the setting of the threshold (λ). Finally, the ranking of states (complications) may be different in some results from the EVAS based on variations in transition probabilities. The variation of all transition parameters was assumed to be in the same range in a state in this study. However, the actual uncertainty of transition probabilities can differ. It will be necessary to consider this in an actual analytical model in future.

## Conclusions

This is the first report on devising a method for the evaluation and ranking of the quantitative value of additional states with uncertainty. It allows cumulative evaluation of transition probabilities, QOL, and cost data of a state using the EVAS. Generally, when we construct a pharmacoeconomic model, we consider states based on transition probabilities (e.g., incidence or mortality rate), but QOL and cost data are also components of a state. This evaluation method is useful because it quantifies the value of a state. In addition, the ranking of additional states in a disease model with multiple complications can be determined to identify which have less impact on a pharmacoeconomic model. The EVAS can therefore take into consideration the value of additional states with the uncertainty of transition probabilities. In the next phase of this continuing study, we plan to demonstrate the relation between the EVAS and pharmacoeconomic evaluation, i.e., the analytic results of interventions, and/or to examine the results in other chronic diseases that have multiple complications, e.g., chronic hepatitis C, etc., because we think that it is necessary to verify that this method will help to construct a simpler analytical model.

## Additional file

Additional file 1: Table S1. Disease type in stroke [20]. **Table S2.** mRS scores for each type of stroke (partly modified) [21] and explanation of each mRS [18,19]. **Table S3.** Breakdown of acute hospital care costs [25,28,29]. **Table S4.** Breakdown of recovery-phase rehabilitation facility care costs [26,29]. **Table S5.** Breakdown of ambulant treatment costs after stroke and CHD [30,31]. **Table S6.** Breakdown of ambulant treatment costs after ESRD [23,29]. **Table S7.** Work lost (4 h) for clinic visit [32]. **Table S8.** Assumed nursing-care costs [27] for each mRS score.
