# Supplementary material for: Expected value of the additional state in evaluating the method of quantification and uncertainty of additional states in an analytical model of grade I hypertension
Source: J Pharm Health Care Sci. 2015 Jan 28;1:3. doi: 10.1186/s40780-014-0006-z (PMC4677729; doi:10.1186/s40780-014-0006-z)
Supplement: Additional file 1: Table S1. — Disease type in stroke [20]. Table S2. mRS scores for each type of stroke (partly modified) [21] and explanation of each mRS [18,19]. Table S3. Breakdown of acute hospital care costs [25,28,29]. Table S4. Breakdown of recovery-phase rehabilitation facility care costs [26,29]. Table S5. Breakdown of ambulant treatment costs after stroke and CHD [30,31]. Table S6. Breakdown of ambulant treatment costs after ESRD [23,29]. Table S7. Work lost (4 h) for clinic visit [32]. Table S8. Assumed nursing-care costs [27] for each mRS score. [file 40780_2014_6_MOESM1_ESM.docx]

Additional file

| Table 1. Disease type in stroke [20].^*^ | |
| --- | --- |
| Disease type | Mean (%) |
| Subarachnoid hemorrhage | 6.8 |
| Cerebral hemorrhage | 17.8 |
| Cerebral infarction | 75.4 |

^＊^The rate of disease type in stroke recurrence is the same.

| Table 2. mRS scores for each type of stroke (partly modified) [21] and explanation of each mRS [18,19 ]. | | | | | | | | |
| --- | --- | --- | --- | --- | --- | --- | --- | --- |
| Stroke type | | | mRS 0 | mRS 1 | mRS 2 | mRS 3 | mRS 4 | mRS 5 |
| Cerebral infarction（%） | | | 45.8 | 22.3 | 6.5 | 6.2 | 9.4 | 9.8 |
| Subarachnoid hemorrhage（%） | | | 12.7 | 21.7 | 13.7 | 11.6 | 26.1 | 14.2 |
| Cerebral hemorrhage（%） | | | 19.0 | 30.8 | 15.5 | 9.4 | 15.7 | 9.5 |
| Modified Rankin scale | | | | | | | | |
| Score | Description | | | | | | | |
| 0 | No symptoms | | | | | | | |
| 1 | No significant disability despite symptoms: able to carry out all usual duties and activities | | | | | | | |
| 2 | Slight disability: unable to carry out all previous activities but able to look after own affairs without assistance | | | | | | | |
| 3 | Moderate disability: requiring some help, but able to walk without assistance | | | | | | | |
| 4 | Moderately severe disability: unable to walk without assistance and unable to attend to own bodily needs without assistance | | | | | | | |
| 5 | Severe disability: bedridden, incontinent, and requiring constant nursing care and attention | | | | | | | |

| Table 3. Breakdown of acute hospital care costs [25, 28, 29]. | |
| --- | --- |
|  | Cost (\) |
| First visit fee (general) | 2,700 |
| Stroke care unit (14 days) (general) | 799,540 |
| Subarachnoid hemorrhage |  |
| Subarachnoid hemorrhage [010020x001x1xx] (43 days) | 1,342,159 |
| Cerebral aneurysm inflow vessel clipping (K176) | 827,300 |
| Artificial respiration (J045) (1 day) | 8,190 |
| Cerebrovascular disease rehabilitation fee I (1 unit: 245 points)  (6.2 units × 43 days) | 653,170 |
| Intracerebral hemorrhage |  |
| Nontraumatic intracranial hematoma [010040x099x00x] (19 days) | 424,860 |
| Cerebrovascular disease rehabilitation fee I (1 unit: 245 points)  (6.2 units × 19 days) | 288,610 |
| Cerebral infarction |  |
| Cerebral infarction [010060x099030x] (18 days) | 553,590 |
| Cerebrovascular disease rehabilitation fee I (1 unit: 245 points)  (6.2 unit × 18 days) | 273,420 |
| CHD (myocardial infarction) (average 25 days) | 3,053,000 |

| Table 4. Breakdown of recovery-phase rehabilitation facility care costs [26, 29]. | |
| --- | --- |
| Rehabilitation care fee | Cost (\) |
| Decubation rehabilitation ward hospitalization fee I  **(1 day: 1,911 points)** (88 days) | 1,681,680 |
| Cerebrovascular disease **rehabilitation fee I**  **(1 unit: 245 points) (6.2 units × 88 days)** | 1,336,720 |

| Table 5. Breakdown of ambulant treatment costs after stroke and CHD [30, 31]. | |
| --- | --- |
|  | Cost of one visit (\) |
| Fees for medical treatment (stroke and CHD) |  |
| Reconsultation fee | 690 |
| Prescription fee | 680 |
| Long-term prescription addition | 650 |
| Specific disease care management fee | 2,250 |
| Ambulant management addition | 520 |
| Fees for dispensing (stroke and CHD) |  |
| Basic dispensing fee | 400 |
| Standard dispensing addition | 300 |
| Dispensing fee | 890 |
| Drug history management administration fee | 410 |
| Drug cost for subarachnoid hemorrhage or intracerebral hemorrhage |  |
| Antihypertensive drug  (valsartan [Diovan] 160 mg/day × 30 days) | 6,600 |
| Drug cost for cerebral infarction |  |
| Antihypertensive drug  (valsartan 160 mg/day × 30 days) | 14,700 |
| Antiplatelet drug  (clopidogrel bisulfate [Plavix] 75 mg/day × 30 days) |  |
| Drug cost for CHD |  |
| First year |  |
| Antihypertensive drug  (valsartan 160 mg/day × 30 days) | 15,000 |
| Antiplatelet drug  (clopidogrel bisulfate 75 mg/day × 30 days) |  |
| Antiplatelet drug (aspirin 100 mg/day × 30 days) |  |
|  |  |
| Second year and thereafter |  |
| Antihypertensive drug  (valsartan 160 mg/day × 30 days) | 6,900 |
| Antiplatelet drug (aspirin 100 mg/day × 30 days) |  |

| Table 6. Breakdown of ambulant treatment costs after ESRD [23, 29]. | |
| --- | --- |
| Treatment | One year (\) |
| Dialysis | 4,910,736 |
| Conservative management | 100,980 |

| Table 7. Work lost (4 h) for clinic visit [32]. | |
| --- | --- |
| Age range (years) | Cost of one visit (¥) |
| 55–59 | 12,229 |
| 60–64 | 9,003 |

| Table 8. Assumed nursing-care costs [27] for each mRS score. | | |
| --- | --- | --- |
| Score | Annual cost (\) |  |
| mRS 0 | － |  |
| mRS 1 | － |  |
| mRS 2 | 1,248,000 |  |
| mRS 3 | 1,989,600 |  |
| mRS 4 | 3,210,000 |  |
| mRS 5 | 4,299,600 |  |
